# The Role of Rho-GTPases and actin polymerization during Macrophage Tunneling Nanotube Biogenesis

**DOI:** 10.1038/s41598-017-08950-7

**Published:** 2017-08-17

**Authors:** Samer J. Hanna, Kessler McCoy-Simandle, Veronika Miskolci, Peng Guo, Michael Cammer, Louis Hodgson, Dianne Cox

**Affiliations:** 1 Department of Anatomy and Structural Biology, Albert Einstein College of Medicine, 1300 Morris Park Ave, Gruss MRRC 306, Bronx, NY 10461 USA; 2 Department of Developmental and Molecular Biology, Albert Einstein College of Medicine, 1300 Morris Park Ave, Gruss MRRC 306, Bronx, NY 10461 USA; 3 Microscopy Core, DART, NYU Langone Medical Center, Bronx, NY 10016 USA; 4 Gruss-Lipper Biophotonics Center, Albert Einstein College of Medicine, Bronx, NY USA; 5 Analytical Imaging Facility, Albert Einstein College of Medicine, Bronx, NY USA

## Abstract

Macrophage interactions with other cells, either locally or at distances, are imperative in both normal and pathological conditions. While soluble means of communication can transmit signals between different cells, it does not account for all long distance macrophage interactions. Recently described tunneling nanotubes (TNTs) are membranous channels that connect cells together and allow for transfer of signals, vesicles, and organelles. However, very little is known about the mechanism by which these structures are formed. Here we investigated the signaling pathways involved in TNT formation by macrophages using multiple imaging techniques including super-resolution microscopy (3D-SIM) and live-cell imaging including the use of FRET-based Rho GTPase biosensors. We found that formation of TNTs required the activity and differential localization of Cdc42 and Rac1. The downstream Rho GTPase effectors mediating actin polymerization through Arp2/3 nucleation, Wiskott-Aldrich syndrome protein (WASP) and WASP family verprolin-homologous 2 (WAVE2) proteins are also important, and both pathways act together during TNT biogenesis. Finally, TNT function as measured by transfer of cellular material between cells was reduced following depletion of a single factor demonstrating the importance of these factors in TNTs. Given that the characterization of TNT formation is still unclear in the field; this study provides new insights and would enhance the understanding of TNT formation towards investigating new markers.

## Introduction

Direct cell contact is an important means of intracellular communication in immune cells in coordinating many functions, for instance the immune synapse between T-cells and antigen-presenting cells^1^. However, contact-dependent communication is not always restricted to immediately adjacent cells. Tunneling nanotubes (TNTs) are thin membranous tubes that connect two cells together and allow for direct cell-cell contact over intermediate distances and can form large networks connecting many cells that can extend cellular communication over larger distances. TNTs are typically thin structures with diameters ranging between 50–800 nm in thickness^2, 3^. TNTs differ from traditional cell contact-dependent signaling in that they can form open channels between cells allowing for the transfer of signaling molecules, soluble proteins, plasma membrane components, vesicles or even organelles^2, 4–6^. TNTs were originally described in cultured rat pheochromocytoma PC12 cells and now are identified in numerous cells types, including almost all immune cells, as long thin F-actin-based membranous channels connecting cells^2^. While all TNTs contain actin, a subset of these structures also contains microtubules, which may account for the increase in diameter in some TNTs^2, 3^. There have been two widely proposed models for TNT formation: actin-driven protrusion or through cell dislodgment, both of which are supported by time-lapse recording studies^2–4, 7–9^. The actin-driven protrusion mechanism involves one cell or both cells extending a protrusion that connect and eventually fuse with the membrane of the other cell^2, 10^. Alternatively, the cell-dislodgement mechanism involves two cells in close contact allowing their membranes to fuse. As cells migrate away from each other, a TNT is formed composed of membrane originating from either one or both cells involved^2, 11^. The precise mechanism of TNT formation is not well understood and may indeed vary depending on the cell type.

Little is known of the signaling pathways that mediate TNT formation, especially in immune cells. One of the first proteins implicated in TNT formation is M-Sec, also known as TNFaip2 (tumor necrosis factor –α-induced protein)^10^. M-Sec has been shown to interact with RalA and the exocyst complex and its expression induces TNT formation in HeLa cells^10^. Further work in HeLa and HEK-293T cells has shown that leukocyte specific transcript 1, LST1, a highly expressed protein in macrophages and DCs, was also required for M-Sec-dependent TNT formation through interaction of RalA with the exocyst complex^12^. It has been proposed that M-Sec and the exocyst complex are involved in supplying membrane that is required for TNT formation.

Despite the apparent universal requirement for F-actin, little has been done to investigate the signaling pathways involved in F-actin generation for TNT formation. It has been proposed that members of the Rho family of GTPases, Rac and Cdc42, involved in actin polymerization, might also be important for TNT formation based on their localization to T-cell TNTs^13^ and Cdc42 inhibition blocked T-cells TNT formation^14^. However, the precise role of Cdc42 in T-cell TNTs was not examined^15, 16^. Recently, the development of new Forster resonance energy transfer (FRET)-based biosensors has allowed the detection of specific spatiotemporal dynamics of Rho GTPases at a high resolution within live single cells, indicating unique functions of these proteins in space and time, which is otherwise difficult with conventional tools. When in the GTP-bound state these GTPases bind and activate downstream effector molecules and regulate multiple cellular functions involving actin polymerization^17–19^. In particular, Cdc42 and Rac1 signal to the downstream regulators WASP and WAVE2 respectively, leading to the activation of the actin nucleator actin-related protein 2/3 (Arp2/3) complex, which has been shown to play roles in multiple macrophage functions including FcγR-mediated phagocytosis, podosome formation and chemotaxis^20–25^. Here, we investigate the role of these actin polymerization regulators during TNT formation in macrophages. Given that actin polymerization is conserved in many cell types, we believe that an analysis of factors regulating actin polymerization in macrophage TNTs would significantly enhance the understanding of TNT formation in general and provide new insights to the field towards investigating new markers for TNTs.

## Results

### Characterization of TNTs in Macrophages

**Figure 1:**
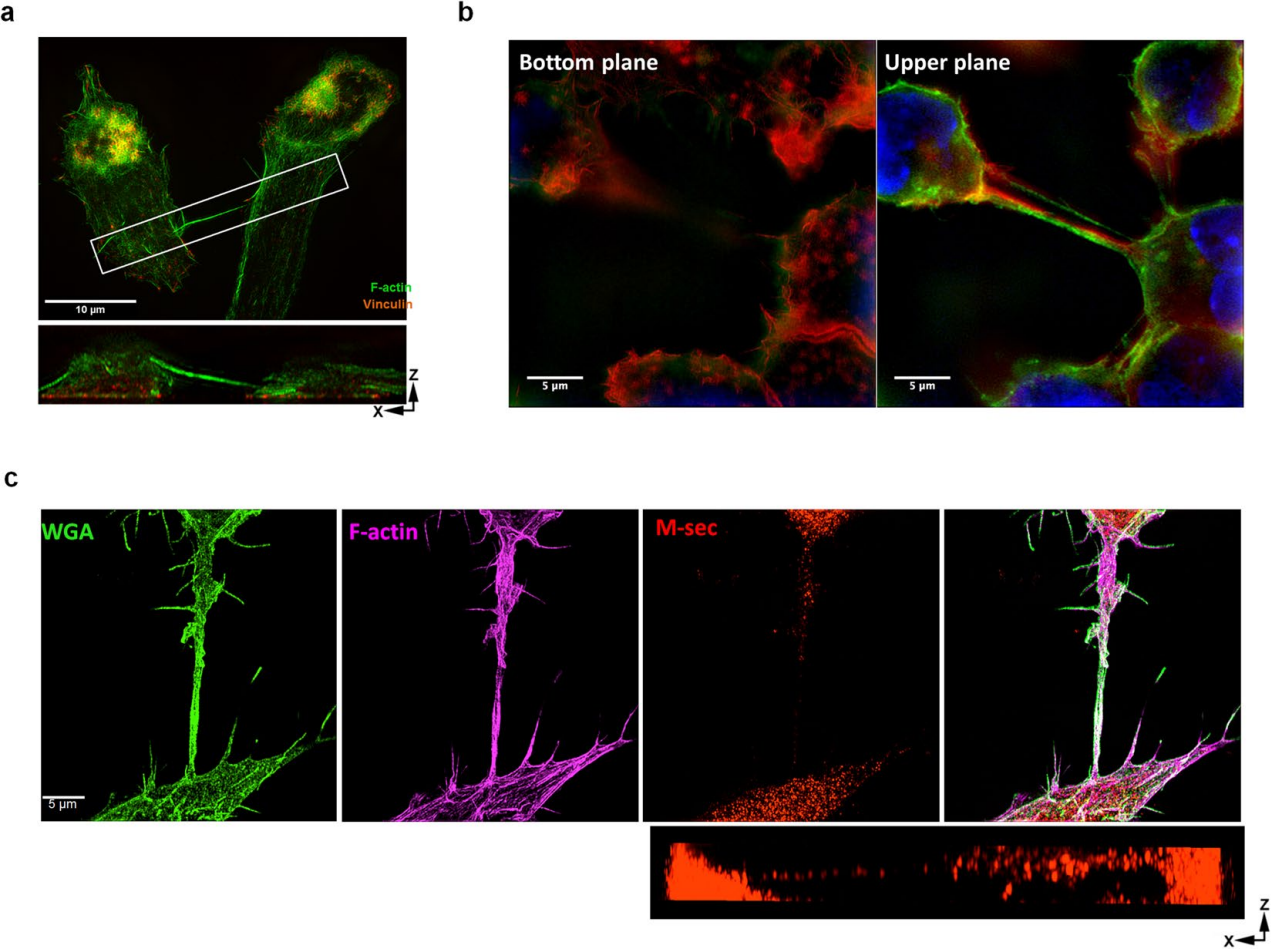
Super-resolution imaging of tunneling nanotubes in RAW/LR5 macrophages. Tunneling nanotubes (TNTs) are identified as long thin actin-containing structures that are above the surface and connecting two cells. (**a**) SIM image of a TNT between two RAW/LR5 macrophages, fixed and stained for F-actin (green) and vinculin (orange). A single TNT (200 nm thick) with F-actin bundle is seen extending from one cell to the other. Orthogonal view shows the xz planes of the boxed area (lower panel), showing a TNT extending above the substrate and connecting two cells. Scale bar = 10 μm. (**b**) SIM image of TNTs connecting RAW/LR5s. Cells are fixed and stained for WGA (green), F-actin (red) and DAPI (blue). Right panel shows the bottom plane where actin-rich structures, podosomes are visible. Left panel shows the upper plane with TNTs connecting multiple cells are visible (see Supplementary movie S4) scale bar = 5 μm. (**c**) Staining for M-sec in TNT between two RAW/LR5 macrophages using 3D SIM. Cells are fixed and stained for WGA (green) to label the membrane and F-actin (magenta) to visualize TNTs and M-sec (red), Lower left panel shows the merged image. Scale bar = 5 μm. Orthogonal view show the xz planes of the same image showing M-sec stain through the TNT.

**Figure 2:**
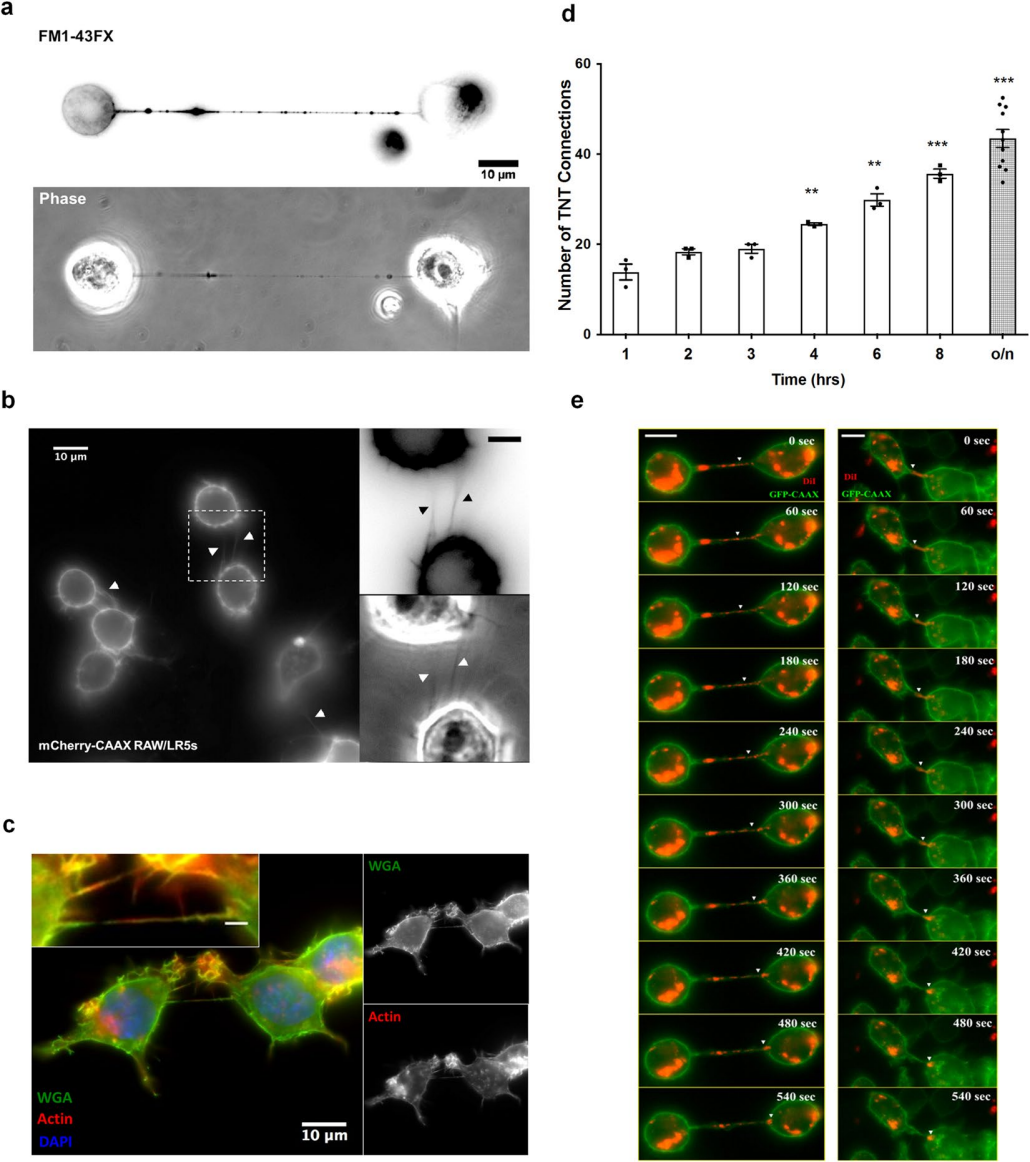
Live cell imaging of tunneling nanotubes in RAW/LR5 macrophages. (**a**) TNTs were identified using live cell imagining of RAW/LR5 macrophages stained with FM1-43FX to visualize the membrane can range from 10 µm to several cell diameters in length. (**b**) Live cell images of TNTs in cells stably expressing mCherry-CAAX plasmid to label the cell membrane as shown. Scale bar in insert = 5 μm. Arrow heads indicate TNTs between cells. Note in both cases the tunneling nanotube (TNT) is connecting two cells and is above the substratum plane. (**c**) Examples of TNTs between RAW/LR5 macrophages in fixed cells stained for WGA (green), F-actin (red) and DAPI (blue). Scale bar in insert = 2 μm imaged under the same conditions as (**b**). (**d**) Time course of TNT formation where cells were quick plated in serum-free RPMI and complete growth media was then added and the time course started. At the indicated time points the media was removed, FM1-43FX stain was added, and TNTs quantified. The number of TNT connections is represented over time (hrs). Dot plot shows the individual values of the number of TNT connections for each independent experiment and the outlined histogram represents the mean average of at least 3 independent experiments for each condition. Error bars +/−SEM with **p < 0.003, ***p < 0004. (**e**) Time-lapse live imaging of DiI-labeled GFP-CAAX RAW/LR5 macrophages showing transfer of DiI-labeled material through TNT connecting two cells. A montage of time-lapse images showing transfer of DiI-labeled material (red) through TNTs connecting GFP-CAAX RAW/LR5 macrophages (green) are shown. Scale bar = 5 µm.

In this study, we characterize the formation of TNTs in macrophages using the RAW/LR5 cell line, a subline of RAW 264.7 cells, which are known to possess functional TNTs^10^. TNTs were defined as long thin structures that contain actin connecting two cells together (TNT connections) as shown in super-resolution imaging using structured illumination microscopy (SIM) in Fig. 1. Importantly, these TNT connections are above the surface and are not attached to the substrate, as shown by a 3D reconstruction of the SIM image in Fig. 1 (see Supplemental movies S1, S2 and S3). Consistent with these structures being membranous nanotubes these actin-containing structures are surrounded by plasma membrane indicated by labeling with fluorescent wheat germ agglutinin (WGA) (Fig. 1b). TNTs appear to lack the adhesion-related protein vinculin (shown in orange in Fig. 1a and Supplemental 3D reconstruction S1), which distinguishes them from actin-rich podosomes that are surrounded by a vinculin ring^22^. In addition, quantitation of SIM images demonstrate that TNT structures have an average diameter of 242 ± 11 nm (n = 10 TNTs). These features of long, thin structures that are above the substrate are consistent with other reports of TNTs. Additionally, a protein known to play a role in macrophage TNTs - M-sec is localized throughout the TNT and appears to be vesicular within the cell body (Fig. 1c and Supplemental 3D reconstruction examples S3 and S4), consistent with its role interacting with the exocyst complex^10, 12^. Therefore, the criteria of long (at least 8 µm) thin structures, above the substrate and connecting two cells were used to identify TNTs throughout the current study. However, these thin structures are known to be very fragile and are often disrupted by fixation and subsequent processing for imaging^4^. In order to minimize TNT breakage we also characterized TNTs in macrophages by live cell imaging. TNT structures were visualized using a membrane dye FM1–43FX that specifically labels the plasma membrane (Fig. 2a). Additionally, RAW/LR5 cell lines were generated to express the fluorescently-tagged plasma membrane markers GFP or mCherry-CAAX which identified TNT structures similar to those observed by immunofluorescence staining with actin and WGA (compare Fig. 2b and c). TNT formation was quantified over time where the percentage of cells that had TNTs with another cell (TNT connections) was determined for a population of cells for each experiment (as described in Methods section). Following cell seeding and attachment in the absence of serum, serum was added and TNTs formed rapidly with a significant increase after 4 hours reaching a plateau by 16 hours (Fig. 2d). To verify that these structures are functional TNTs we confirmed that vesicular material was trafficked between TNT connected cells. Live time-lapse imaging using GFP-CAAX labeled RAW/LR5s which were also labeled with DiI demonstrated the transfer of labeled material through TNTs (Fig. 2e and see Supplemental movies S5 and S6). Overall, these data indicate that these structures are bona fide TNTs and demonstrate the criteria used to identify TNTs in macrophages.

### Actin Polymerization is required for TNT formation

**Figure 3:**
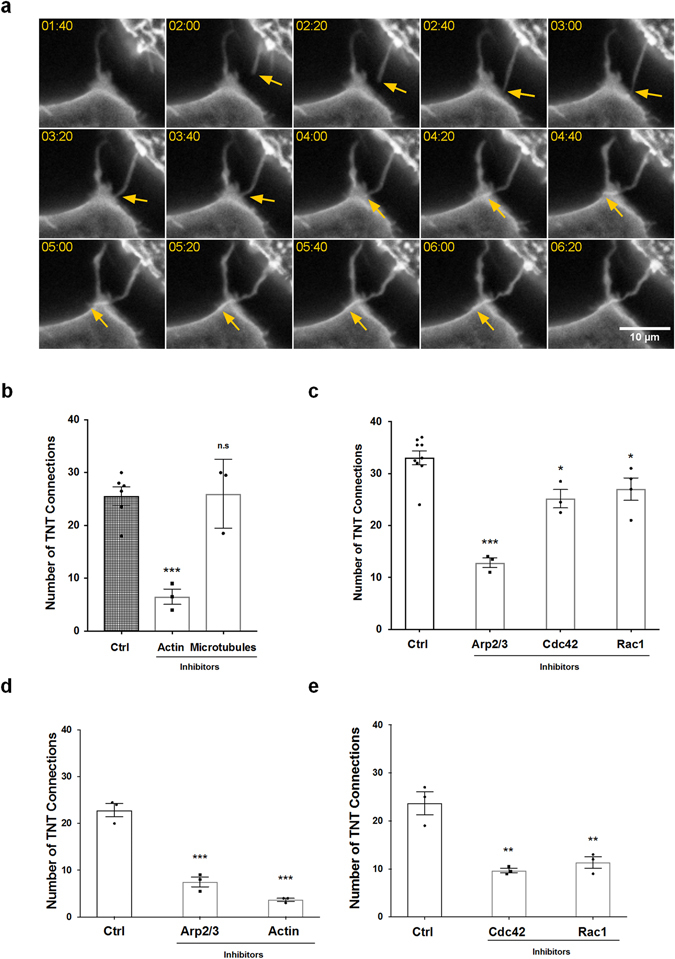
Actin polymerization signaling pathways are required for TNT formation in RAW/LR5 macrophages. (**a**) Montage of time-lapse live images of GFP-CAAX RAW/LR5 macrophages showing the formation of a TNT-like protrusion (indicated by the yellow arrow) that then connects to an adjacent cell. For visualization of fine less intense structures within the narrow dynamic range available for display, images were local contrast enhanced. Scale bar: 10 µm. (**b**) Following quick adherence, RAW/LR5 macrophages were incubated with growth media containing vehicle control, actin polymerization inhibitor cytochalasin D (2 μM), or microtubule polymerization inhibitor Nocodazole (2 μM). TNT formation was quantified 4 hours after treatment. The number of TNT connections is represented as in (2d). (**c**) Following quick adherence, RAW/LR5 macrophages were incubated with growth media containing vehicle control, Arp2/3 inhibitor CK666 (40 μM), Cdc42 inhibitor ML-141 (10 μM) or Rac1 inhibitor 6-thio-GTP (10 μM). TNT formation was quantified 4 hours after treatment. (**d**) Following quick adherence, bone marrow derived macrophages (BMMs) isolated from 3 independent mice were treated with vehicle control, Arp2/3 inhibitor CK666 (40 μM), or actin polymerization inhibitor cytochalasin D (2 μM). TNT formation was quantified 4 hours after treatment. (**e**) Following quick adherence, BMMs isolated from 3 independent mice were treated with vehicle control, Cdc42 inhibitor ML-141 (10 μM) or Rac1 inhibitor 6-thio-GTP (10μM). TNT formation was quantified 4 hours after treatment. Data in all graphs is represented as dot plots showing the individual values of the number of TNT connections for each independent experiment in each case. The outlined histograms represent the mean average of at least 3 independent experiments. Error bars +/−SEM with *p < 0.05, **p < 0.02, ***p < 0.003, ns: not significant.

In previous studies using other cell types, TNTs have been shown to form by two mechanisms: actin-driven protrusion and/or cell dislodgement. Time lapse imaging of RAW/LR5 cells show the rapid formation of long thin “TNT-like” precursor structures that are above the substrate, curved, and can be branched (See Supplemental movie S7) consistent with TNTs in other cell types. In addition, time lapse imaging of RAW/LR5 cells expressing GFP-CAAX show active TNT-like protrusions that lead to full TNT formation between two cells (Fig. 3a and see Supplemental movie S8). This suggested that actin-driven protrusions could generate TNTs. Previous studies demonstrated that in standard tissue culture conditions RAW/LR5 macrophages are not extremely motile in the absence of a chemoattractant^22^. This taken together with the visual evidence of a protrusion-based mechanism suggests that the majority of TNTs in our cultures are formed by actin-driven protrusion.

To determine potential pathways that may be important in formation of TNTs, inhibitors of various pathways were added after cell attachment. A four-hour time point was chosen to allow for sufficient TNT formation, inhibitor uptake and inhibitory activity, but little inhibitor degradation. Control experiments determined the effective concentrations and incubation times of the inhibitors by examining other macrophage functions including Cdc42-dependent podosome formation^22, 26^ and Rac1-dependent CSF-1-induced actin ruffling^20, 27, 28^ (Supplemental Fig. S1 and S2). First, we confirmed the requirement for the structural components, actin and microtubules, for biogenesis in macrophages. Cells were allowed to attach prior to treatment with the inhibitors cytochalasin D and nocodazole, which led to the inhibition of barbed-end actin polymerization and microtubule assembly, respectively. Inhibition of microtubule assembly did not decrease the number of TNT connections formed suggesting that while microtubules may be present in some portion of TNTs they were not required for formation (Fig. 3b). Consistent with other reports, inhibition of actin polymerization severely decreased TNT formation (Fig. 3b). However, complete inhibition of TNT formation was not seen when actin polymerization was inhibited. This may be due to formation of TNTs during the time cells were rapidly attached to surface before the uptake and activation time of the inhibitor. Next, the importance of factors mediating actin polymerization on TNT formation was tested starting with the well-known actin nucleation factor, Arp2/3. TNT formation was significantly decreased when the Arp2/3 actin nucleation factor was inhibited with the Arp2/3 specific inhibitor CK666 (Fig. 3c). It should be noted that inhibition of the Arp2/3 complex did not yield the same decrease in TNT formation as cytochalasin D inhibition of actin polymerization (50% inhibition compared to 75% inhibition). Alternatively, this result may indicate the potential role of other methods of actin polymerization such as formins. We believe this may be due to potential differences in inhibitor uptake, stability, and/or mechanism of actions. Also, while the time allowed for TNT formation was normalized in all experiments, there may be slight differences in inhibitor effectiveness at 4 hours. Similar results were also seen when using primary bone marrow derived macrophages (BMMs) (Fig. 3d and e), indicating an important role for the Arp2/3 complex in TNT biogenesis.

### Role of Rho GTPases Rac1 and Cdc42 in regulating TNT biogenesis

**Figure 4:**
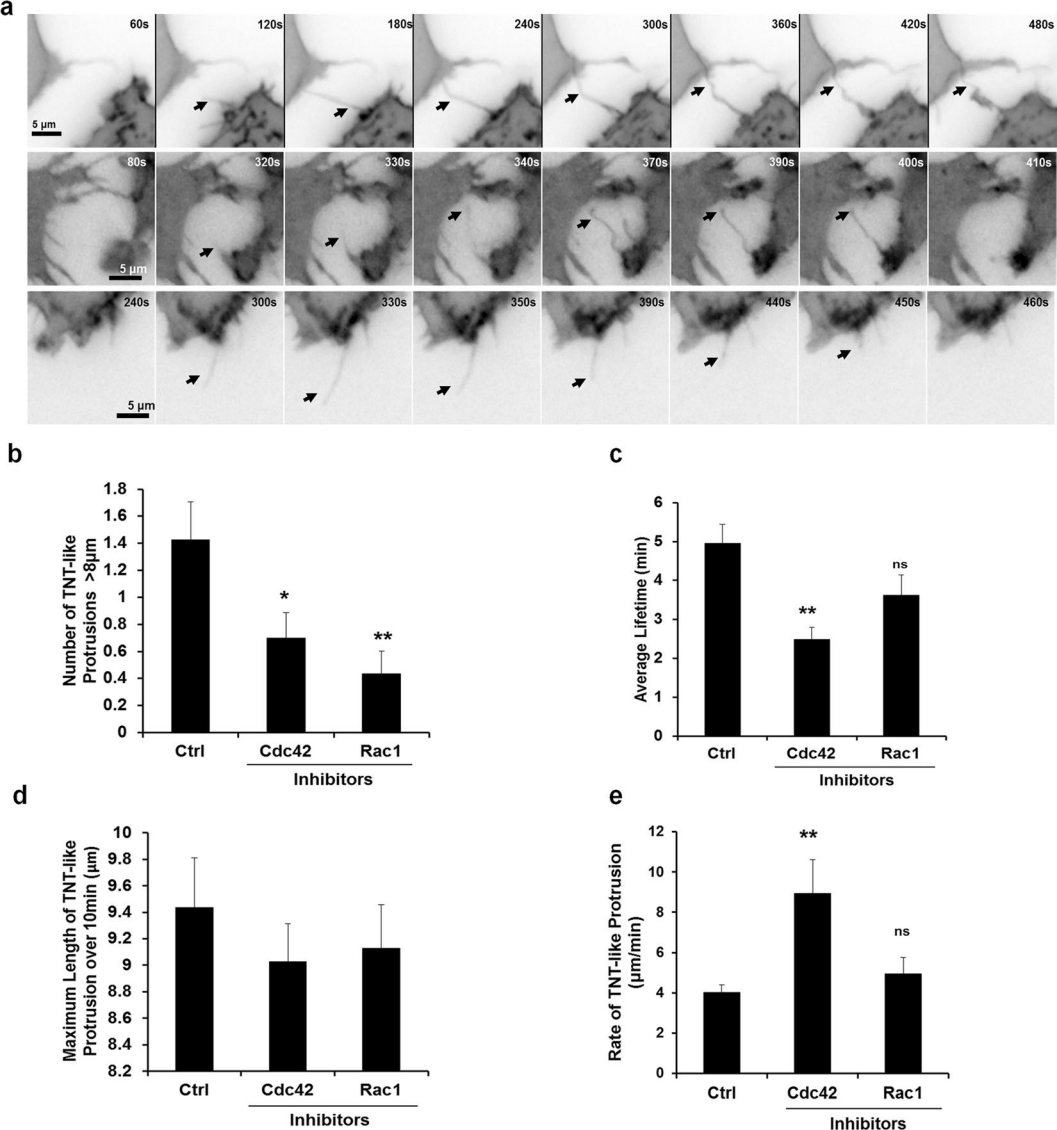
Roles of Cdc42 and Rac1 during TNT formation. (**a**) Following quick adherence, GFP-CAAX RAW/LR5 macrophages were incubated with growth media containing vehicle control, Cdc42 inhibitor ML-141 (10 μM) or Rac1 inhibitor 6-thio-GTP (10 μM). Time-lapse live imaging of GFP-CAAX RAW/LR5 macrophages in the presence of DMSO (upper panels), Cdc42 (middle) or Rac1 (bottom) inhibitors in BWD buffer imaged over 10 min periods. Arrow heads indicate when ‘TNT-like’ protrusions initiate. Scale bar: 5 µm. (**b**) Quantitation of the number of TNT-like protrusions (>8 µm in length) per cell. (**c**) Quantitation of the average duration of TNT-like protrusions (>8 μm) over the course of the time-lapse images (10 min). (**d**) Quantitation of the maximum length of TNT-like protrusions and (**e**) the TNT-like protrusion rate (maximum length/time it takes to reach the maximum length). Data represents the mean average of at least 25 cells. Error bars +/−SEM with *p < 0.05, **p < 0.02, ns: not significant.

Cdc42 and Rac1 are two different Rho GTPases well known to regulate Arp2/3-dependent actin polymerization. To determine if these Rho GTPases were involved in TNT formation, their activity was inhibited using specific inhibitors. Decreasing Cdc42 activity using the Cdc42 specific inhibitor ML-141 resulted in a partial but significant 25% decrease in TNT formation in both RAW/LR5 and primary BMMs (Fig. 3c and e). Decreasing Rac1 activity using 6-thio-GTP^29^ also decreased TNT formation to a similar extent as seen when Cdc42 was inhibited. This suggested that both Cdc42 and Rac1 contribute to TNT formation. To further explore the roles of these two Rho GTPases in TNT biogenesis we performed live cell time-lapse imaging using GFP-CAAX expressing RAW/LR5 cells. We analyzed the formation and dynamics of TNTs in the absence and presence of Cdc42 or Rac1 inhibitors (Fig. 4a and Supplemental movies S9–S11). Results showed that inhibiting either Cdc42 or Rac1 significantly reduced the number of TNT-like protrusions (>8 μm) as compared to vehicle control (Fig. 4b). Additionally, detailed analysis of TNT-like protrusions from time-lapse images revealed that the lifetime of single TNT-like protrusions was reduced when either Rho GTPase was inhibited and in particular when Cdc42 activity was blocked (Fig. 4c). Yet, there was no significant difference in the maximum length of the TNT-like protrusions within the monitored time interval (10 min). However, while inhibition of Cdc42 reduced the lifetime of TNT-like protrusions, there was an actual increase in protrusion rate after Cdc42 inhibition as compared to vehicle control (Fig. 4d and e). In addition, while there was a significant reduction in the number of TNT-like protrusions observed following Rac1 inhibition, the effect on life-time or rate of protrusions was not significantly different from control treated cells. These results suggested that while both Cdc42 and Rac1 were involved in formation of TNT-like precursors, they may be playing distinct roles.

**Figure 5:**
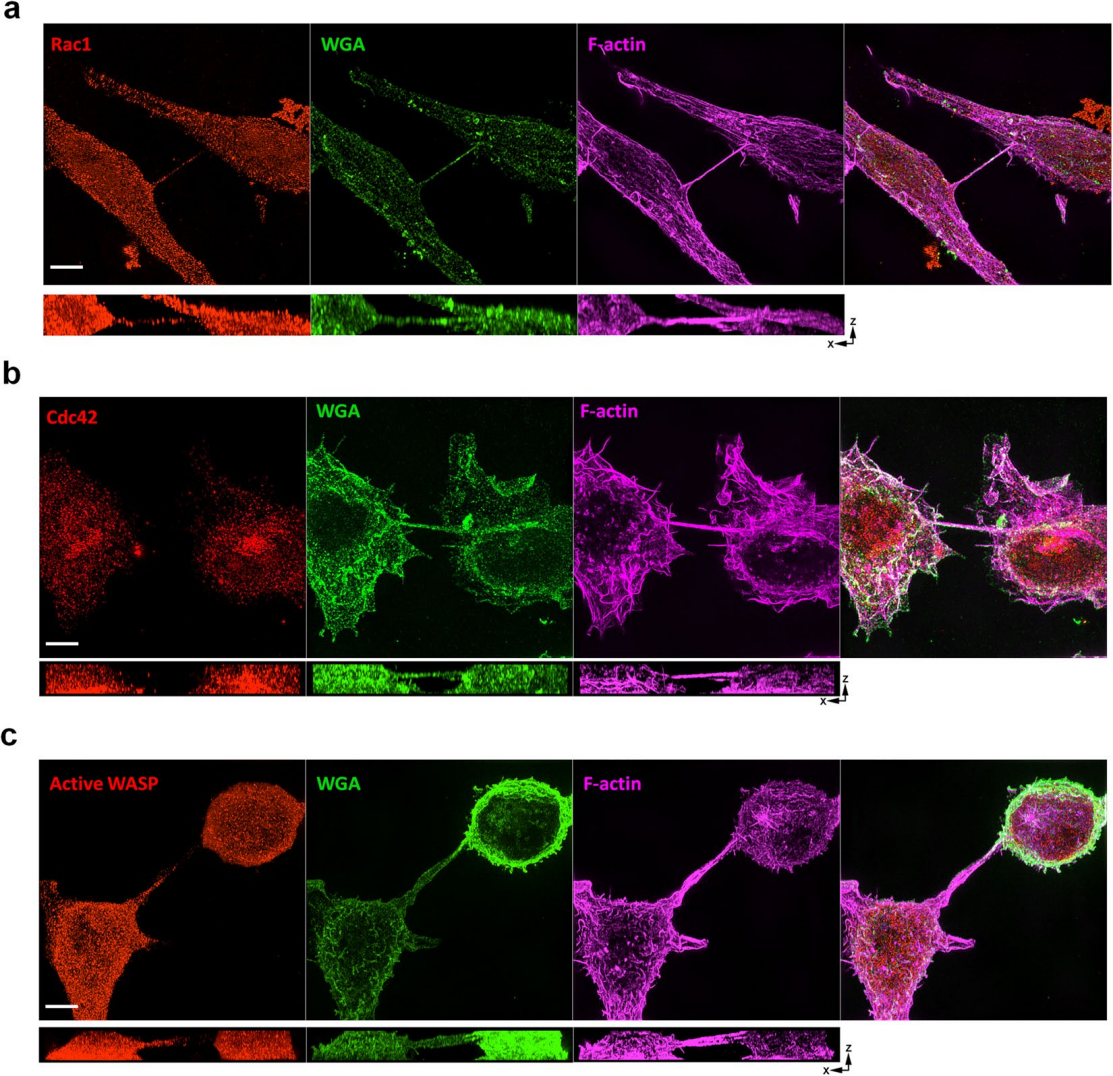
Localization of Rac1, Cdc42, downstream active WASP and in RAW/LR5 macrophage TNTs. 3D SIM images of TNTs between RAW/LR5 macrophages fixed and stained for WGA (green) and F-actin (magenta) to label the TNTs. (**a**) Co-staining for Rac1 (red) shows localization throughout the TNT connecting two RAW/LR5 macrophages. (**b**) Staining for Cdc42 (red) shows very little within the TNT. (**c**) Staining for activated WASP using a conformation-sensitive antibody CSA (red) downstream of Cdc42 shows localization within the TNT. For all images scale bars = 5 µm and orthogonal views show the xz planes for each of the separate channels.

**Figure 6:**
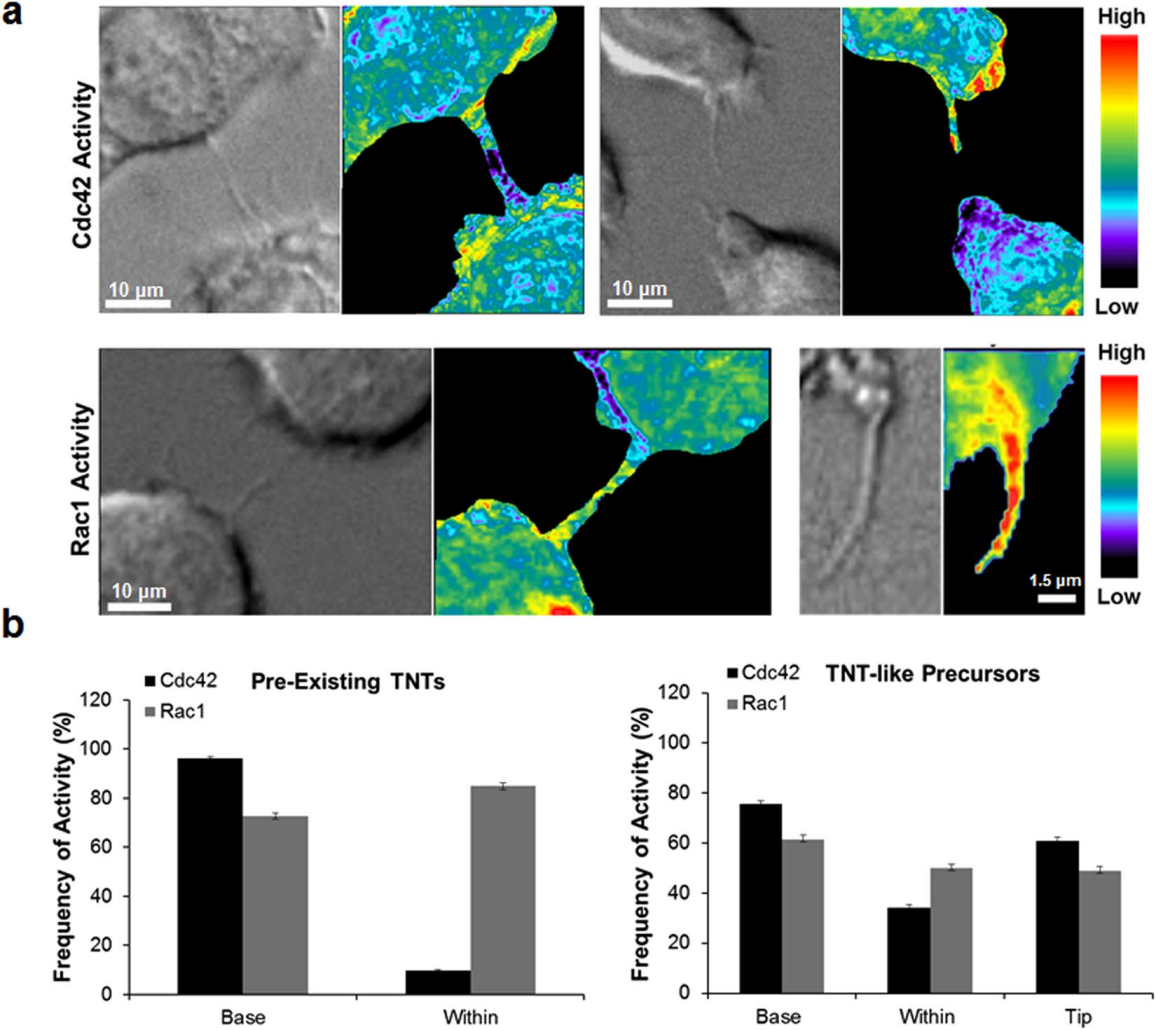
Cdc42 and Rac1 activity during TNT formation in RAW/LR5 macrophages. (**a**) Representative images of time-lapse ratiometric imaging of RAW/LR5 macrophage cell line with inducible expression of Cdc42 biosensor in pre-existing TNTs (left panels) or TNT-precursors (right panels). Scale bars = 10 µm. (**b**) Representative images of time-lapse ratiometric imaging of RAW/LR5 macrophage cell line with inducible expression of Rac1 biosensor in pre-existing TNTs (left panels) or TNT-like precursors (right panels). Scale bars: Left = 10 µm, Right = 1.5 µm. (**c**) Quantitation of the frequency of Cdc42 activity (black bars) or Rac1 activity (grey bars) in the base and within pre-existing TNTs (left graph) or at the base, within and at the tip of growing TNT-like precursors (right graph). Data is represented as an average of at least 20 TNT structures. Error bars represent +/−SEM.

To further explore the different roles of these two Rho GTPases during TNT biogenesis, we first analyzed their localization within TNTs formed between two cells using 3D-SIM imaging. Interestingly, despite the equal requirement for Rac1 and Cdc42 in TNT formation, Rac1 was localized throughout the TNT itself, while Cdc42 showed minimal localization in the TNTs (Fig. 5a and b). We next looked at the Cdc42 downstream effector WASP, using a conformation-sensitive antibody (CSA) that detects the activated fraction of endogenous WASP/N-WASP with a higher affinity to WASP^30^. It should be noted that this antibody was originally generated against a region only accessible when WASP is activated by Cdc42^31^. Given the fact that WASP is expressed at 15 times the level of N-WASP in RAW/LR5 cells^32^, this suggests that this reagent can be used to determine the activation state of endogenous WASP. Our results show that active WASP localizes throughout the TNT connecting two macrophages (Fig. 5c) and suggests that once activated by Cdc42, WASP become incorporated into the actin structure of the TNT. Since staining only shows the localization of these Rho GTPases at a fixed time and when TNTs are already formed, we then investigated the activation status of both Cdc42 and Rac1 in pre-existing TNTs as well as in TNT-like precursor structures over time using our previously published FRET-based biosensors^24, 27, 33^. Surprisingly, despite both Rho GTPases being equally important for macrophage TNT formation, we observed differential activation patterns for Cdc42 and Rac1 (Fig. 6). In pre-existing TNTs (Fig. 6a, left panels), Cdc42 activity was predominantly concentrated at the base of the TNTs, and was excluded from within the TNT itself as only present less than 10% of the time (Fig. 6b). This result is consistent with the lack of Cdc42 in the TNT observed by SIM (Fig. 5b). Unlike the case with pre-existing TNTs, Cdc42 activity was present throughout the TNT-like precursor and appeared to be concentrated in the tip (Fig. 6a and b, right panels), which is in keeping with the localization of activated WASP (Fig. 5c). In contrast to Cdc42, Rac1 activity seemed to be distributed throughout the structure in the pre-existing TNTs (Fig. 6a and b, left panels). This was also true when analyzing TNT-like precursors where there was no significant difference in the distribution of Rac1 activity between the base, within or at the tip (Fig. 6b). Collectively, these data suggest that these Rho GTPases are differentially localized and activated in both TNT-like precursors and pre-existing TNTs consistent with a differential role in TNT biogenesis.

### Role of downstream effectors WASP and WAVE2 in regulating TNT biogenesis

**Figure 7:**
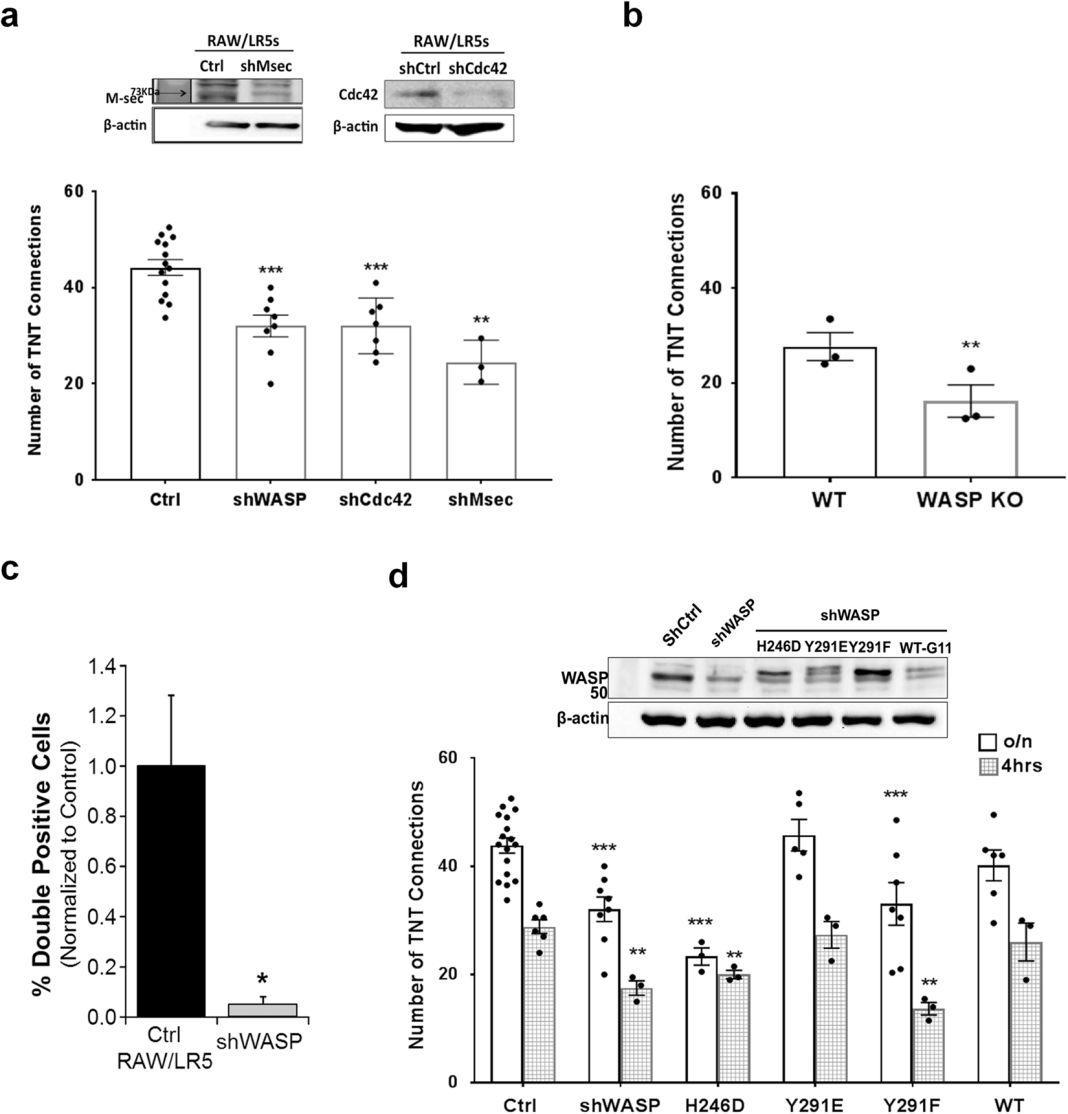
Actin nucleation promoting factor WASP and its regulation are required for TNT formation. (**a**) Control RAW/LR5 macrophages or cells with stably reduced levels of WASP, Cdc42, or M-sec proteins using shRNA constructs. Western blot analysis shows the corresponding expression levels of M-sec (left) and Cdc42 (right) of the cell lines, β-actin is used as a loading control. Cells were plated in complete growth medium and TNTs were allowed to form overnight before quantitation. Data is represented as in the previous figures and represents the mean average of at least 3 independent experiments for each case. Error bars +/−SEM with **p < 0.02, ***p < 0.003. (**b**) TNT formation was quantified in WT bone marrow derived macrophages (BMMs) or WASP Knockout (KO) BMMs isolated from 3 independent mice. Error bars +/−SEM with *p < 0.01. (**c**) Dil-labeled cells were co-cultured with GFP containing cells for 24 hours. Transfer of DiI labeled material was quantitated using Flow cytometry and the % cells that have transferred DiI material (double positive cells) is shown. (**d**) Control RAW/LR5 macrophages, shWASP, and shWASP cells expressing mutant forms of WASP: a Cdc42-binding deficient (H246D) mutant, a phospho-mimetic (Y291E) mutant, a phospho-deficient (Y291F) mutant or a Wild-type (WT) form of WASP were used to quantify TNT formation. Upper panel shows western blot analysis for the expression levels of the corresponding cell lines used; β-actin is used as a loading control. TNT formation in the corresponding cell lines was quantitated overnight (clear bars) and at 4hrs (gray pattern bars) following quick plating. Data is presented as dot plots of values from individual experiments and as outlined histograms of the mean average of at least 3 independent experiments. Error bars +/−SEM with *p < 0.05, **p < 0.01, ***p < 0.001.

Arp2/3 activity is well known to be regulated by Cdc42 through WASP. In order to study the effect of WASP on TNT formation, we used RAW/LR5 cell lines previously generated where either WASP or Cdc42 protein levels were reduced by shRNA^22, 34^. Since the half-life of inhibition is not an issue with stable shRNA unlike with the use of inhibitors, we examined the constitutive level of TNTs with these cell lines. A greater that 90% reduction of WASP levels led to a significant decrease in the number of cells connected by TNTs, similar to that found following reduction of Cdc42 (Fig. 7a). To determine how this phenotype compared to other proteins that have been previously identified to regulate TNT formation, we reduced endogenous expression levels of M-Sec^10^. Consistent with previous studies, we observed a decrease in TNT formation with the loss of M-Sec. Furthermore, this decrease was of a similar degree to that observed following WASP or Cdc42 reduction (Fig. 7a). Similar results were also obtained when BMMs from wild type and WASP-deficient mice were compared (Fig. 7b). To determine the significance of the role of WASP in TNT functionality we used a standard assay for TNTs^3, 12, 35, 36^ by monitoring transfer of DiI labeled material from donor cells into GFP expressing recipient cells by determining the percentage of double positive cells by flow cytometry. Transfer of material occurred in control RAW/LR5 macrophages but was completely abolished in WASP-deficient cells (Fig. 7c). Interestingly, this almost complete block in transfer occurred despite only a partial decrease in TNTs in shWASP cells. These experiments suggest that while structures resembling TNTs are present in WASP-deficient cell cultures, these structures are not functional in regards to vesicle transfer (Fig. 7c). TNT formation was also monitored at 4 hours, the time used in the inhibitor studies, so that both formation and stability could be monitored. At both time points, cells that have been depleted of WASP had significantly less TNTs than control cells (Fig. 7d) suggested that WASP plays a role in TNT formation that results in long term effects. The efficacy of protein reduction in the shMsec and shCdc42 cell lines was confirmed (Fig. 7a) and the level of WASP in the control cell line was compared to that of the shWASP cell line and the lines reconstituted with mutant forms of WASP (Fig. 7d). Taken together, these data demonstrate that the actin polymerization pathway initiated by Cdc42 activation of WASP was involved in TNT formation as shown by the decrease in TNT formation when this pathway is inhibited.

The activation of WASP in macrophages requires the binding of Cdc42 inducing a conformational change in WASP that permits interaction with Arp2/3^37, 38^. In addition, our group has previously reported that tyrosine phosphorylation of WASP downstream of Cdc42 activation was also important during regulation of numerous macrophage functions including phagocytosis, chemotaxis, podosome dynamics, and matrix degradation^22, 39, 40^. We then examined TNT formation in shWASP cells stably expressing wild type (WT) human WASP and signaling mutations in WASP including Cdc42-binding deficient (H246D), phospho-deficient WASP (Y291F) or phospho-mimetic (Y291E) mutant versions. Cells expressing either the WT or phospho-mimetic (Y291E) mutant form of WASP were able to form TNTs to a similar extent as regular RAW/LR5 cells (Fig. 7d). However, cells expressing a Cdc42-binding deficient (H246D) showed a significant decrease in TNT formation. In addition, the phospho-deficient (Y291F) form of WASP did not form TNTs to the same extent as control cells, but behaved similarly to the shWASP cells. This trend was present when either constitutive levels of TNTs were examined (Fig. 7d, clear bars) or when looking at an earlier time point in formation (4 hours, Fig. 7d, patterned bars). We have also previously shown that phosphorylation of WASP occurs in a Src kinase-dependent manner^30, 39^. In fact, reducing Src kinase activity using the inhibitor PP2 also decreased TNT formation to a similar extent as seen with the Rho GTPase inhibitors (23% ± 6.54 p = 0.02). Overall, these data in conjunction with the localization of activated WASP (Fig. 5C) demonstrates that regulation of WASP activity is important in the formation and stabilization of TNTs.

**Figure 8:**
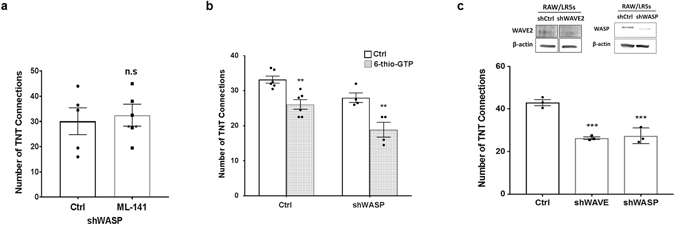
Both the WASP pathway and the Rac1 pathway contribute to TNT formation. (**a**) Control RAW/LR5 macrophages and shWASP cells were incubated in growth media containing vehicle control or the Cdc42 inhibitor ML-141 (10 μM). After 4 hours the number of TNT connections formed was quantified. (**b**) Control RAW/LR5 macrophages and shWASP cells were incubated in growth media containing vehicle control (black bars) or the Rac1 inhibitor 6-thio-GTP (10 μM) (gray pattern bars). After 4 hours the number of TNT connections formed was quantified. (**c**) RAW/LR5 macrophages were transiently transfected with either non-targeting or shRNA constructs against WASP or WAVE proteins overnight and then selected with puromycin. Representative western blot analysis of the relative expression levels of WASP (left) and WAVE (right); β-actin is used as a loading control. Selected cells were plated in MatTek dishes and TNTs were allowed to form overnight and then quantified and plotted as in previous figures for at least 3 independent experiments. Error bars +/−SEM with *p < 0.05, **p < 0.02, ***p < 0.003, ns: not significant.

While both Cdc42 and Rac1 were important during TNT formation (Fig. 3c and e), only a partial decrease in TNT formation was observed when inhibiting either GTPase, which was not as large as seen when Arp2/3 was inhibited. This data suggested that multiple activators of Arp2/3 may contribute to TNT formation. To assess whether Cdc42 and WASP inhibition has an additive effect, Cdc42 was inhibited in shWASP cells. Our results show a non-significant difference of 0.56% ± 2.87 (p = 0.736) (Fig. 8a), confirming that WASP and Cdc42 activity occur in the same pathway. To assess if both the Cdc42 and Rac1 pathways are involved and to limit inhibitor toxicity, Rac1 activity was inhibited in shWASP cells (Fig. 8b). When both Rac1 and WASP activities were reduced, an approximate 50% decrease in TNT formation was observed, similar to that observed when inhibiting Arp2/3 (Fig. 8b compared to Fig. 3c). Since inhibiting both pathways had an additive effect on TNTs, we determined which Rac1 downstream effector was involved in TNT formation. Rac1 is well known to regulate the WAVE family proteins and we have previously identified that WAVE2 is the major family member expressed in macrophages^41^. Depletion of WAVE2 was achieved by transient shRNA expression rather than using stable cell lines due to the transient effectiveness of the shRNA^22, 41^. To appropriately match conditions a transient knockdown of WASP was also performed as an appropriate comparison for WAVE^42^. Fewer constitutive TNTs were observed after overnight culture in cells with reduced levels of either WASP or WAVE (Fig. 8c) with similar decreases in TNTs in either WAVE (38%) and WASP (36%) reduced cells suggesting both pathways are equally as important to TNT formation. It should be noted that transient reduction of WASP levels led to a slightly larger decrease in TNT formation than observed with stable shWASP cells, likely due to the greater level of protein reduction seen in transient versus stable knockdown protocols^22^. This data indicated that loss of the WASP/WAVE family members of Arp2/3 nucleating factors decreased the number of TNT connections, suggesting that multiple means of Arp2/3 activation are involved. These results reveal a novel role for both WASP and WAVE2 in macrophage TNT formation.

## Discussion

A wide variety of cell types have been observed to frequently interact via TNTs^43^. While the function of TNTs can vary among cell types, all known TNTs, particularly in immune cells, can mediate the transfer of information either by cargo or signals from one cell to another (reviewed in ref. 44). Despite the commonly known functions of TNTs, their biological significance is widely variable depending on the cell type. The importance of TNT-mediated communication is just beginning to be understood. However, very little is known about the formation of these structures other than the two widely proposed models for TNT formation: actin-driven protrusion and a cell-dislodgment mechanism^2–4, 7–9^. Our findings demonstrate that the formation of TNTs, particularly in macrophages, is dependent on actin polymerization factors. This involves the Rho family GTPases Rac1 and Cdc42, and their downstream effectors WAVE and WASP respectively. Moreover, our study demonstrates an important role for WASP in both formation and function of TNTs. This suggests an additional role for WASP in macrophage cell-cell communication. Macrophages derived from Wiskott-Aldrich Syndrome (WAS) patients are defective in chemotaxis and are unable to directionally migrate towards CSF-1^45^. Therefore, WASP may be playing a role in coordination of cell-cell signaling and communication, in addition to its known roles in macrophage functions such as phagocytosis and chemotaxis. This suggests that WASP plays a central role in regulating macrophage functions.

Previously, our group has extensively studied the Rho GTPases Rac1 and Cdc42 in multiple macrophage functions including phagocytosis, chemotaxis and podosome formation^22, 24, 27^. We have observed an additive effect on TNT formation when inhibiting both the Rac1 and Cdc42 pathways, suggesting that they act within different pathways during the formation of TNTs. This is in accordance with previous known functions of Rac1 and Cdc42 and downstream effectors in actin-rich protrusions^20, 27^, where they play different but coordinated roles. Traditionally, Cdc42 is thought to be important for the formation of spike-like filopodia, whereas Rac1 is involved in the formation of broad meshwork of branched actin-rich lamellipodia^46^. Filopodia are classically regarded as sensors which extend from the cell to detect chemotactic cues critical for direction sensing during cell migration^47^. It has been previously reported that in neuronal cells, TNTs form as protrusions similar to filopodia that require the filopodial motor myosin X ^48^. Using time-lapse live cell imaging, we have observed that TNTs also form by extending filopodia-like protrusions from one cell, or both cells that intertwine and eventually fuse (Supplemental movies S7 and S8). This is similar to previous studies done in PC12 cells where the interplay of filopodia-like protrusions between two cells can act as precursors for TNTs^49^. However, TNTs connecting two cells are known to be distinct from filopodia in many respects. This includes that TNTs do not need to be attached to the substrate where many filopodia are commonly found. TNTs can also be curved or branched, and can mediate transfer of membrane and vesicular material from one cell to another^7, 11, 44, 50^.

FRET-based biosensors have become very powerful tools to monitor both the localization and activation of the dynamic Rho family GTPases. Our genetically-encoded, single-chain, FRET-based Cdc42 and Rac1 biosensors are applicable for both fixed- and live-cell imaging^24, 33^. Importantly, our design maintains an intact C-terminal polybasic region of the Rho GTPases. This allows proper native intracellular localization and interaction with upstream regulators. Using our FRET-based biosensors, we were able to investigate the activation patterns of both Cdc42 and Rac1. Our results showed that Cdc42 was activated mostly at the base and excluded from within pre-existing TNTs (Fig. 6). This is similar to previous studies looking at the activation of Cdc42 in filopodia where Cdc42 activity was seen at the base of the filopodia, flanking the actin core^51^. Similarly, during TNT formation Cdc42 was activated at the base and at the tip of growing TNT-like precursors (Fig. 6). Moreover, while the events of branching TNTs observed in our live cell imaging were too infrequent during live cell imaging to quantify, Cdc42 appeared to be activated at branch points in the precursors. Consistent with a role for Cdc42 in TNT formation, inhibition of Cdc42 reduced the number of TNT-like precursors. Interestingly, these TNTs-like precursors formed at a faster rate, yet displayed a significantly reduced lifetime with a slight decrease in the maximum length. These results suggest that Cdc42 may be required for the initial formation and persistence of TNTs potentially through the activation of its downstream effector WASP. This corresponds to previous work done in our group looking at Cdc42 activity during the formation of podosomes, actin-rich adhesive and matrix degrading structures formed in macrophages. Using the Cdc42 FRET-biosensor we observed that Cdc42 was activated only transiently at the initial step during podosome formation^24^. This initial activation of Cdc42 may be required for WASP activation of Arp2/3. We have also shown previously that in response to chemotactic stimulus, in the absence of WASP membrane protrusions still occurred and reached the same maximum length, but displayed delayed onset, increased protrusion rate and failed to persist as long as control cells^40^. Unlike Cdc42, Rac1 seemed to be activated throughout the length of the forming TNT-precursor and this activity was maintained in formed TNTs (Fig. 6), suggesting that Rac1 activity may be required for TNT maintenance. Furthermore, upon Rac1 inhibition cells exhibited significantly less TNT-like protrusions with no significant effect on duration, maximum length or rate of formation (Fig. 4). We have also observed that the onset of Rac1 and Cdc42 is different during the formation of random protrusions in macrophages^27^. Therefore, while Cdc42 and Rac1 are both critical modulators of the actin cytoskeleton dynamics, our data adds to this and suggests that these Rho GTPases play obligate, albeit different roles during the formation of TNTs in macrophages.

Our findings are supported by the limited previously published data. Cdc42, Rac1, ezrin and N-WASP were all localized to TNTs, but the requirement of these factors in TNT formation was not assessed^13^. Treatment of Jurkat T-cells with the Cdc42 specific inhibitor secramine A blocked TNT formation^14^. These studies support our findings that the Cdc42 and Rac1 pathways are involved in TNT formation. In another study, using an artificial system to produce TNTs in HeLa cells by the over-expression of M-Sec, inhibition of Cdc42 by expression of dominant negative construct resulted in decreased TNT formation, but Rac1 inhibition showed no effect^10^. These results may differ from ours due to the artificial nature of TNT formation in this study as HeLa cells are not known to constituently make TNTs, or there are differences in the mechanism of TNT formation in different cell types. Future studies are needed to explore the roles of these Rho GTPases in other cell types that have been characterized to constitutively form TNTs.

Many different biological functions have been proposed for TNTs that are widely variable depending on the cell type. However, they appear to be important in immune cell functions and for the coordination of immune responses^44^. For example, Schiller *et al*. hypothesized that TNTs allow the transfer of antigenic information between dendritic cells involving the transfer of MHC class I molecules^12^. Another study in dendritic cells demonstrated that TNTs can facilitate intercellular antigen exchange resulting in enhanced antigen-specific T-cell responses^52^. Other studies have shown that TNTs can mediate receptor-ligand interaction such as in NK cell TNTs with target cells. These TNTs contain a submicron scale junction that accumulates the activating receptor NKG2D and its signaling adaptor DAP10 and the NKG2D activing protein MHC class I chain-related protein A (MICA) expressed on the surface of infected cells, which resulted in an enhanced rate of target cell lysis compared to conventional cytolytic synapse^53^. Furthermore, work from the Cherqui group showed that TNTs can mediate the transfer of cystinosin-containing lysosomes in a bi-directional manner between wild type hematopoietic stem cell derived macrophages and cystinotic fibroblasts *in vitro* and into kidney tubular cells *in vivo* and consistent with the long-term kidney preservation observed in Ctns−/− mice following bone marrow transplantation^54, 55^. These studies suggest the potential of TNTs in physiologic functions.

Interestingly, patients with WAS often develop autoimmune diseases in addition to their primary immune deficiencies. It has been speculated by others that this is due to a breakdown in tolerance or due to defective clearance by innate immune cells and reduced clearance of apoptotic material^56^. Moreover, WASP-dependent actin remodeling has been suggested to regulate innate activation of dendritic cells by inhibiting the TLR-IFNα pathway through specific intracellular trafficking and compartmentalization of TLR-9 ligands. However, given the role of WASP and actin polymerization in TNT formation and function, it is possible that some of the immune dysregulation in these patients maybe be due to TNT defects. TNTs have also been observed within other cell types, particularly in tumor cells^57^, where it has been suggested that TNTs play an important role in tumor microenvironments mediating the transfer of cellular contents between tumor cells and non-malignant stromal cells^58, 59^. More importantly, a recent study provided evidence of similar membranous structures connecting astrocytoma tumor cells *in vivo* which results in the formation of a functional multicellular network that correlates with prognostic features of malignant brain tumors^60^. We have previous demonstrated that WASP is important in the paracrine interaction between macrophages and breast carcinoma cells that mediates tumor cell invasion and metastasis^61^. Our study suggests that given the role of WASP in TNT formation and function, TNTs might also be involved in direct cell-cell communication between macrophages and tumor cells. This topic is currently under investigation within our group and extends the potential biological relevance of TNTs within different biologically relevant conditions. This highlights the importance of the current study in understanding the mechanisms of TNT formation in general which will provide new insights to the field towards investigating new markers for TNTs.

## Methods

### Cell lines, transfections and plasmids

Murine RAW/LR5 monocyte/macrophages^8^ were maintained at 37 °C in a 5% CO^2^ incubator and were cultured in RPMI 1640 medium (Mediatech, Inc.) supplemented with 10% heat-inactivated newborn calf serum (Sigma) and antibiotics (100 units/ml penicillin, 100 µg/mL streptomycin, Sigma). The cell lines (RAW/LR5, RAW/LR5 shWASP, RAW/LR5 shWASP cells stably expressing wild type human WASP, phospho-deficient WASP (Y291F) or phospho-mimetic (Y291E) and RAW/LR5 shCdc42) were all previously described^20, 22, 39^. In some cases, protein levels were reduced using transient transduction of RAW/LR5 cells with short hairpin RNA (shRNA) constructs directed against WAVE and WASP proteins. RAW/LR5 cells infected with pSuper.retro.puro plamids expressing control, WASP-targeting shRNA, or WAVE-targeting shRNA have been described previously^22^. Membrane-labeled RAW/LR5s were generated by stable transfection using DNA construct containing GFP-CAAX plasmid cloned into a pcDNA3.1 vector^25^. Following transfection with Fugene HD cells were selected in the presence of G418 (Invitrogen).

### TNT Quantitation

TNT formation was monitored by rapidly attaching RAW/LR5 cells to MatTek dishes in RPMI for 30 minutes to minimize TNT formation while allowing for adherence to the plate (400,000 cells per 35 mm dish). Then growth media was added and TNTs were allowed to form for 4 hours (the exception being the time course experiment in which cells incubated for indicated times). Cells were briefly washed with Buffer with Divalent Cations (BWD) and then labeled and imaged using the vital membrane dye FM1-43FX according to manufacturer’s instructions (Thermo Fisher Scientific). The number of TNT was quantified as follows, in order to be counted as TNT connection at least one membranous structure must be present connecting two cells, at least partially non-adherent to the substratum and having a minimum length of 8 µm. Cells with no TNTs must be within one cell body length of another cell without touching any other cell to be counted as negative. A total of 128 cells were quantified for every experiment and experiments were repeated at least 3 times. The data was presented as the individual values of the number of TNT connections for each independent experiment. When inhibiting proteins during formation, inhibitors where added to the growth media and introduced to cells after rapid attachment. Actin polymerization inhibitor Cytochalasin D (Sigma) was used at 2 µM. Microtubule inhibitors Nocodazole (Sigma) was used at 2 µM. Arp2/3 inhibitor CK666 (Abcam) was used at 40 µM. Cdc42 inhibitor ML-141 was used at 10 µM and Rac 1 inhibitor 6-thio-GTP (Jena Bioscience) was used at 10 µM. For overnight incubations, 100,000 cells were plated onto MatTek dishes in growth media and allowed to incubate overnight. 128 cells were counted in each condition for each experiment.

### Immunofluorescence and structural illumination microscopy (SIM)

Cells plated in MatTek dishes or on 1.5 glass coverslips were fixed in 3.7% formaldehyde in BWD buffer (20 mM HEPES, 125 mM NaCl, 5 mM KCl, 1 mM KH_2_PO_4_, 5 mM glucose, 10 mM NaHCO_3_, 1 mM MgCl_2_, 1 mM CaCl_2_, pH 7.4) for 7 minutes at 37 °C, to visualize TNTs, cells were then stained with Wheat Germ Agglutinin, Alexa Fluor 488 conjugate (Molecular Probes) at a concentration of 1ug/ml for 10 minutes at room temperature. Cells were then permeabilized in 0.2% Triton X-100 (in BWD) for 5 minutes. F-actin was visualized by staining with Alexa Fluor 647-labelled phalloidin (Molecular Probes). Coverslips were mounted in SlowFade Diamond Antifade mountant (Molecular Probes). Multichannel structured illumination microscopy (SIM) images were acquired using a Nikon Structured Illumination N-SIM system on an inverted Nikon ECLIPSE Ti-E equipped with a 100 × 1.49 NA objective. Multicolor fluorescence was generated using diode lasers (488, 561 and 647 nm). Acquisition was performed with electron-multiplying CCD cameras (Andor iXon3 DU897) 512 × 512 pixel frame size. Z stack images were imaged with a step size of 0.12 µm. Three reconstruction parameters (Illumination Modulation Contrast, High Resolution Noise Suppression and Out of Focus Blur Suppression) were extensively tested to generate consistent images across experiments without abnormal features or artifacts and producing the best Fourier transforms. The images were processed using Nikon Elements software. 3D reconstruction was performed with Imaris software. For vinculin staining, cells were blocked with 10% milk in TBS followed by incubation with a specific monoclonal antibody (hVin1, Sigma) followed by incubation with Alexa Fluor 488-labelled goat anti-mouse IgG (Molecular Probes). F-actin was visualized by staining with Alexa Fluor 568-labelled phalloidin (Molecular Probes). Coverslips were mounted in PBS and imaged using DeltaVision OMX Blaze 3D-SIM super-resolution microscope (Applied Precision, GE Healthcare) with lasers at 488 and 568 nm, an Olympus 100X N.A. 1.40 UPLSAPO oil objective with immersion oil of refractive index 1.516 or 1.518, and Evolve EMCCD cameras (Photometrics). Fifteen raw images per plane at Z axis intervals of 0.125 µm were taken at three angles with five phases. Structured Illumination microscopy reconstructions and image registration were performed using OMX SoftWorx calibrated and maintained by the Bio-Imaging Resource Center at the Rockefeller University. Analysis performed with ImageJ (Rasband, W.S., ImageJ, U. S. National Institutes of Health, Bethesda, Maryland, USA, http://imagej.nih.gov/ij/, 1997–2016).

### Transfer of labeled material

To test functionality of TNTs cells were stained with DiI (Invitrogen) according to the manufacturer’s instructions. Then these labeled cells were incubated with RAW/LR5 cells expressing GFP at a 1:1 ratio overnight. Cells were washed three times with PBS, detached from plate, and analyzed for transfer of DiI material into GFP positive cells using a FACS Calibur flow cytometer. Control experiments when DiI labeled cells were fixed before co-culture showed no transfer of labeled material suggesting the transfer of material is not due to phagocytic activity of recipient cells. For live-cell imaging, GFP-CAAX RAW/LR5 cells were labeled with DiI (Invitrogen) for 15 min. Cells were then washed 1x with PBS and plated in MatTek dishes at 100,000 cells per dish and incubated overnight in growth medium. The MatTek dish was then mounted on a heated stage maintained at 37 °C for time-lapse imaging. To image DiI transfer through TNTs, imaging was performed in RPMI supplemented with 5% FBS. Images were acquired in 10 seconds intervals for 10 minutes using Olympus 60X/1.40 N.A. oil objective of an Olympus X 71 microscope coupled to a Sensicam cooled CCD camera. Analysis of captured images was performed using ImageJ software.

### Live-cell imaging and TNT dynamics analysis

TNT formation was monitored by rapidly attaching GFP-CAAX labeled RAW/LR5 cells to MatTek dishes in RPMI for 30 minutes (400,000 cells per 35 mm dish). Then growth media was added and TNTs were allowed to form for 4 hours in the presence of DMSO (vehicle control), Cdc42 inhibitor ML-141 at 10 µM or Rac1 inhibitor 6-thio-GTP (Jena Bioscience) used at 10 µM. After, 4 hours of incubation, the MatTek dish was then mounted on a heated stage maintained at 37 °C for time-lapse imaging. To image TNTs, imaging was performed in BWD supplemented with 5% FBS. Following temperature stabilization, images were acquired 10 seconds intervals for 10 minutes at 60X magnification. Analysis of captured images was performed using Metamorph and ImageJ softwares. “TNT-like” precursors were identified as cellular extensions that are off the substrate and reach a minimum length of 8 µm within the time of imaging (10 min). Measurement of protrusion length over time was done using the segmented line tool on Metamorph and the data was exported to Excel (Microsoft) for statistical analysis. TNT-like protrusion rate was measured as maximum length/time it takes to reach maximum length.

### Live-cell imaging and FRET analysis

Stable macrophage cell line RAW/LR5 used previously in ref. 27 with inducible expression of Cdc42 or Rac1 biosensors were used for live cell imaging. Repression of biosensor expression was maintained by culturing cells in RAW medium supplemented with 2 µg/ml doxycycline (MP Biomedicals). Induction of biosensor expression was performed as previously described in). Briefly, 48 hours prior to imaging, cells were washed with PBS to remove doxycycline medium then trypsinized and replated at low density. For optimal induction of biosensor expression, cells were cultured in RAW medium supplemented with 10% tetracycline-free serum. After 24 hours, cells were briefly trypsinized and replated at low density for another 24hrs. Then cells were lifted again and plated in 35 mm MatTek dish at a concentration of 2 × 10^5^ overnight. The MatTek dish was then mounted on a heated stage maintained at 37 °C during imaging. To image TNTs, imaging was performed in BWD supplemented with 5% FBS to achieve optimal signal-to-noise ratio. Following temperature stabilization, images were acquired 10 s intervals for 10 min at 60X magnification. Both FRET and mCer1 emission channels were acquired simultaneously at each frame using two side-mounted cameras in order to eliminate motion artifacts as described in ref. 62, followed by an acquisition of the differential interference contrast (DIC) channel at the third camera mounted on the bottom port of the microscope. mVen emission was captured to confirm biosensor expression at first frame only to minimize photobleaching during imaging session. Detailed descriptions of live-cell imaging, microscope settings and image processing are provided in refs 62 and 63. FRET/donor ratiometric analysis at every frame was performed using Metamorph and MatLab software. GTPase activity was determined in an area representing the base, within or the tip of each TNT structure, then compared to area within the cell body. Frequency of GTPase activity was then determined over ≥10 frames.

### Data analysis

For every experiment n value was greater or equal to 3 independent experiments (n ≥ 3). Results were considered statistically different when two-tailed analysis was performed using a Student t-test resulted in differences between two means with a p value of less than or equal to 0.05 (α ≤ 0.05). Error bars signify standard error of the mean (+/−SEM).

## Electronic supplementary material

Movie ﻿S1:3D reconstruction showing the xz planes of the boxed area in Figure 1a.

Movie ﻿S2: 3D reconstruction showing the Z-stack images of the Figure 1b.

Movie ﻿S3: 3D reconstruction of Figure 1c showing TNT connecting RAW/LR5 macrophages.

Movie ﻿S4: 3D reconstruction of another example showing TNT connecting RAW/LR5 macrophages

Movie ﻿S5: Example 1 of time-lapse of DiI-labeled GFP-CAAX RAW/LR5 cells showing the transfer of DiI-labeled between the two connected cells.

Movie ﻿S6: Example 2 of time-lapse of DiI-labeled GFP-CAAX RAW/LR5 cells showing the transfer of DiI-labeled between the two connected cells.

Movie ﻿S7: Time-lapse of DIC image of RAW/LR5 cells showing the formation of a TNT-like precursor protruding from either cell and eventually intertwine and fuse to form a TNT.

Movie ﻿S8: Time-lapse of GFP channel of GFP-CAAX RAW/LR5 cells showing the formation of a TNT-like precursor protruding from one cell to the adjacent one to form a TNT.

Movie ﻿S9: Time-lapse of GFP channel and corresponding DIC image of GFP-CAAX RAW/LR5 cells in the presence of DMSO vehicle control.

Movie ﻿S10: Time-lapse of GFP channel and corresponding DIC image of GFP-CAAX RAW/LR5 cells in the presence of Cdc42 inhibitor.

Movie ﻿S11: Time-lapse of GFP channel and corresponding DIC image of GFP-CAAX RAW/LR5 cells in the presence of Rac1 inhibitor.

Supplementary Information
