# Supplementary material for: The Role of Rho-GTPases and actin polymerization during Macrophage Tunneling Nanotube Biogenesis
Source: Sci Rep. 2017 Aug 17;7:8547. doi: 10.1038/s41598-017-08950-7 (PMC5561213; doi:10.1038/s41598-017-08950-7)
Supplement: Supplementary file 12 — Supplementary Information [file 41598_2017_8950_MOESM12_ESM.pdf]

The Role of Rho-GTPases and actin polymerization during Macrophage Tunneling Nanotube Biogenesis

Samer J. Hanna[Φ1], Kessler McCoy-Simandle[Φ1], Veronika Miskolci[1], Peng Guo[1,4,5], Michael Cammer[3], Louis Hodgson[1,4], and Dianne Cox*[1,2,4]

[Φ] These authors contributed equally
* Corresponding author
[1]Department of Anatomy and Structural Biology
[2]Department of Developmental and Molecular Biology
Albert Einstein College of Medicine
1300 Morris Park Ave, Gruss MRRC 306
Bronx, NY 10461, USA
[3] Microscopy Core, DART, NYU Langone Medical Center, NY, NY 10016
[4] Gruss-Lipper Biophotonics Center, Albert Einstein College of Medicine, Bronx, NY
[5]Analytical Imaging Facility, Albert Einstein College of Medicine, Bronx, NY

Author Information:
Samer J. Hanna, MS
samer.hanna@phd.einstein.yu.edu
Kessler Mccoy-Simandle, PhD,
k.mccoy-simandle@ucc.edu
Correspondence should be addressed to:
Dianne Cox, PhD,
dianne.cox@einstein.yu.edu

Supplemental Figure Legends:

Figure S1: Dose dependent effect of Rac1 inhibitor (6-thio-GTP) on actin response. RAW/LR5s were starved for 2 hours in RPMI medium, then pre-treated with DMSO control or increasing concentrations of Rac1 inhibitor as indicated for 30 minutes before the end of the starvation period. Cells were then stimulated with CSF-1 (20 ng/ml) for 3 minutes at 37°C before fixation and staining for F-actin. F-actin intensity was then quantified for each condition and normalized to unstimulated control. Data represents the mean average of 3 independent experiments with at least 32 cells quantified in each experiment. Error bars +/- SEM with ***$p<0.0005$, ns: not significant.

Figure S2: Dose dependent effect of Cdc42 inhibitor (ML-141) on podosome formation. RAW/LR5 cells were pre-treated with DMSO control or increasing concentrations of Cdc42 inhibitor as indicated. Cells were then treated with Cytochalasin D (CytoD) for 15 minutes to dissolve the preexisting podosomes and allow for the synchronous reformation of podosomes (as in [22]). CytoD was then washed and replaces with fresh medium and cells were incubated at 37°C before fixation and staining for F-actin (green) and Vinculin (red) as markers for podosomes. The percentage of cells with podosomes was then quantified and represented in comparison to untreated control. Data represents the mean average of 3 independent experiments with at least 25 cells quantified in each experiment. Error bars +/- SEM with * $p<0.05$, ***$p<0.003$, ns: not significant.

Supplemental Movie Legends:

Movie S1: 3D reconstruction showing the xz planes of the boxed area in Figure 1a. Structured Illumination microscopy reconstructions and image registration were performed using the OMX SoftWorx software calibrated and maintained by the Bio-Imaging Resource Center at the Rockefeller University. Analysis performed with ImageJ (Rasband, W.S., ImageJ, U. S. National Institutes of Health, Bethesda, Maryland, USA, http://imagej.nih.gov/ij/, 1997-2016).

Movie S2: 3D reconstruction showing the Z-stack images of the Figure 1b with step size = 0.12 µm. Z-stack shows the bottom plane and the upper plane of the image showing the presence of the TNTs connecting RAW/LR5 macrophages in the upper plane of the image. Cells are stained with WGA (green) to label the membrane, F-actin (red) and DAPI (blue). Images were acquired using a Nikon Structured Illumination N-SIM system on an inverted Nikon ECLIPSE Ti-E equipped with a 100× 1.49 NA objective. Analysis performed with ImageJ (Rasband, W.S., ImageJ, U. S. National Institutes of Health, Bethesda, Maryland, USA, http://imagej.nih.gov/ij/, 1997-2016).

Movie S3: 3D reconstruction of Figure 1c showing TNT connecting RAW/LR5 macrophages and stained with WGA (green) to label the membrane, F-actin (magenta) and M-sec (red). Images were acquired using a Nikon Structured Illumination N-SIM system on an inverted Nikon ECLIPSE Ti-E equipped with a 100× 1.49 NA objective. 3D reconstruction was generated using Imaris software calibrated and maintained by the Analytical Imaging Facility at Albert Einstein College of Medicine.

Movie S4: 3D reconstruction of another example showing of TNT connecting RAW/LR5 macrophages and stained with WGA (green) to label the membrane, F-actin (magenta) and M-sec (red). Images were acquired using a Nikon Structured Illumination N-SIM system on an inverted Nikon ECLIPSE Ti-E equipped with a 100× 1.49 NA objective. 3D reconstruction was generated using Imaris software calibrated and maintained by the Analytical Imaging Facility at Albert Einstein College of Medicine.

Movie S5: Example 1 of time-lapse of DiI-labeled GFP-CAAX RAW/LR5 cells showing the transfer of DiI-labeled between the two connected cells. Duration of original sequence: 10 min. Magnification 60X, 2X2 binning. Frame interval: 10 s. Scale bar = 5 µm.

Movie S6: Example 2 of time-lapse of DiI-labeled GFP-CAAX RAW/LR5 cells showing the transfer of DiI-labeled between the two connected cells. Duration of original sequence: 10 min. Magnification 60X, 2X2 binning. Frame interval: 10 s. Scale bar = 5 µm.

Movie S7: Time-lapse of DIC image of RAW/LR5 cells in BWD showing the formation of a TNT-like precursor protruding from either cell and eventually intertwine and fuse to form a TNT. Duration of original sequence: 10 min. Magnification 60X, 2X2 binning. Frame interval: 10 s. Scale bar = 10 µm.

Movie S8: Time-lapse of GFP channel of GFP-CAAX RAW/LR5 cells in BWD showing the formation of a TNT-like precursor protruding from one cell to the adjacent one to form a TNT. Image sequences were analyzed using the CLAHE plugin for ImageJ with a local neighborhood of 35 pixels for the contrast enhancement. Duration of original sequence: 10 min. Magnification 60X, 2X2 binning. Frame interval: 10 s. Scale bar = 10 µm.

Movie S9: Time-lapse of GFP channel and corresponding DIC image of GFP-CAAX RAW/LR5 cells in BWD in the presence of DMSO vehicle control showing the formation of a TNT-like precursor protruding from one cell to the adjacent one to form a TNT. Duration of original sequence: 10 min. Magnification 60X, 2X2 binning. Frame interval: 10 s. Scale bar = 10 µm.

Movie S10: Time-lapse of GFP channel and corresponding DIC image of GFP-CAAX RAW/LR5 cells in BWD in the presence of Cdc42 inhibitor M-141 (10 µM) showing the formation of a TNT-like precursor protruding from one cell to the adjacent one to form a TNT. Duration of original sequence: 10 min. Magnification 60X, 2X2 binning. Frame interval: 10 s. Scale bar = 10 µm.

Movie S11: Time-lapse of GFP channel and corresponding DIC image of GFP-CAAX RAW/LR5 cells in BWD in the presence of Rac1 inhibitor 6-thio-GTP (10 µM) showing the formation of a TNT-like precursor protruding from one cell. Duration of original sequence: 10 min. Magnification 60X, 2X2 binning. Frame interval: 10 s. Scale bar = 10 µm.

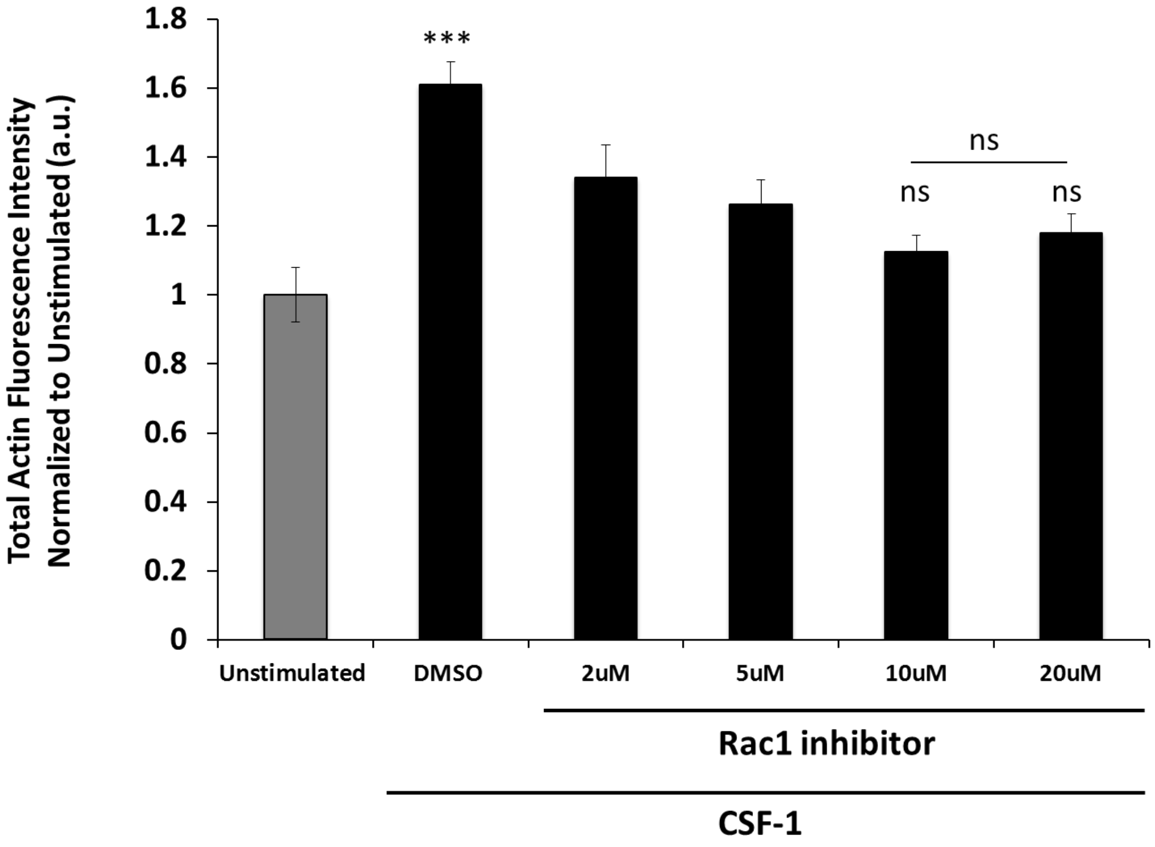

Supplementary Figure S1

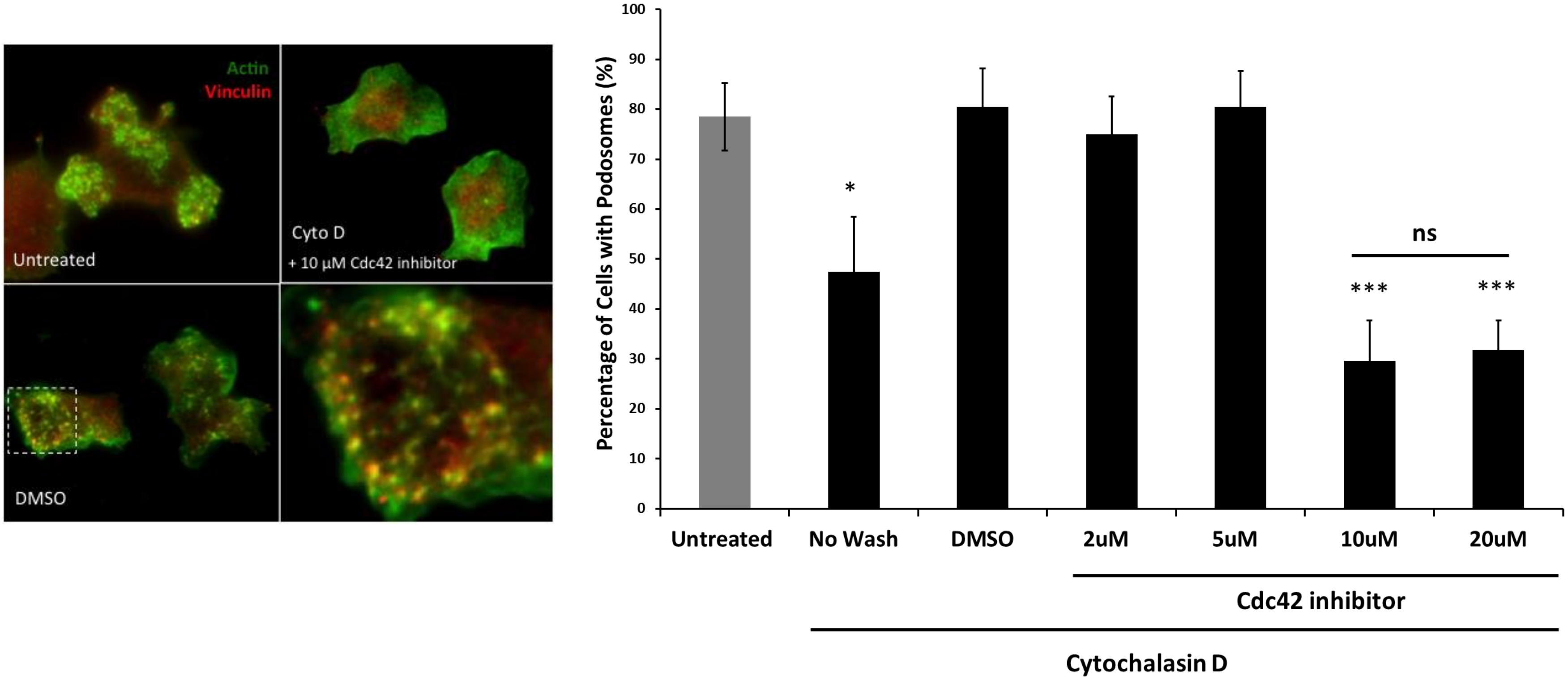

Supplementary Figure S2
